# The effectiveness of acupoint catgut embedding in hyperlipidemia with obesity: Protocol for a systematic review and meta-analysis

**DOI:** 10.1097/MD.0000000000020342

**Published:** 2020-05-29

**Authors:** Peipei Hong, Yang Gao, Qiuyue Wang, Xianliang Qiu, Qiu Chen

**Affiliations:** Hospital of Chengdu University of Tradional Chinese Medicine, Sichuan, China.

**Keywords:** acupoint catgut embedding, hyperlipidemia, meta-analysis, obesity, protocol, systematic review

## Abstract

**Background:**

Hyperlipidemia is a common disease characterized as blood lipid metabolism disorders, which is always companied with obesity. Hyperlipidemia is the leading cause of the cardiovascular disease (CVD) closely associated with mortality, and the risk can further elevate in the obese population. Additionally, it induces stroke and acute pancreatitis. Studies demonstrated that acupoint catgut embedding is an effective therapy for hyperlipidemia with obesity. Whereas, there is no systematic review and meta-analysis to support the point. Thus, we intend to conduct a systematic review and meta-analysis to testify its effectiveness in hyperlipidemia with obesity.

**Methods:**

We will include articles by searching the following database: PubMed, Embase, Web of Science, Cochrane Controlled Trials Register (CENTRAL), China National Knowledge Infrastructure (CNKI), China Biomedical Literature Database (CBM), Chinese Science Journal Database (VIP), and Wanfang database. Whats more, the manual search can be executed as the complement of database searching. Endnote X8 and RevMan V.5.3 will be used to complete the process of study selection, data analysis, as well as date management.

**Result:**

The primary outcomes contain the reduction of TC, TG, LDL-C, HDL-C, and BMI, body weight (WB), waist circumference(WC), body fat percent (F%) from baseline to the end of studies. The second outcome is the number of adverse events during the total trial.

**Conclusion:**

We will summarize sufficient evidence to confirm the therapeutic effect and safety of acupoint catgut embedding in hyperlipidemia with obesity.

**INPLASY registration number:**

INPLASY202040036.

## 1 Introduction

Hyperlipidemia is a metabolic disorder disease, which is manifested by a decrease in serum high- density lipoprotein (HDL) or an increase in serum total cholesterol (TC), triglyceride (TG) and low-density lipoprotein (LDL).^[1,2]^ In recent years, due to changes in individuals lifestyle and dietary structure, the number of patients with hyperlipidemia has soared all over the world. In China, the overall prevalence rate of hyperlipidemia is about 30.9% in military officers,^[3]^ while the overall pooled-prevalence of total dyslipidemia was estimated at 25.3% in children and adolescents.^[4]^ Additionally, the prevalence in Russia is up to 45%.^[5]^ Cardiovascular disease (CVD) is the leading cause of death. Hyperlipidemia, as one of the pathological bases of atherosclerosis, significantly increases the risk of cardiovascular and cerebrovascular diseases.^[6,7]^ Besides, it also poses a threat to acute pancreatitis.^[8]^ Hyperlipidemia is usually companies with a sequence of metabolic diseases, especially obesity. Hyperlipidemia participates in obesitys development. Similarly, obesity can induce lipide metabolism disorder. Besides, obesity is also regarded as an independent risk factor for cardiovascular disease.^[9]^ Therefore, Hyperlipidemia with obesity sufferers a higher risk for the development of CVD. Additionally, search indicates obesity and hypertriglyceridemia significantly increase the risk for peripheral neuropathy on early-stage diabetes, independent of glucose control.^[10]^ Consequently, targeted lipid-lowing treatment should be an emphasis on the prevention and treatment of CVD, stroke, acute pancreatitis, and the improvement of life quality.

Deeply affected by diet and lifestyle, we considered dietary control and sustained exercise as the cardinal invention measure of dyslipidemia. Therefore, individuals with dyslipidemia have to adhere to life and diet interventions, regardless of drugs are used or not. But it can not maintain stable efficacy and is not enough for hyperlipidemia.^[11]^ Therefore, it is essential to combine with drug therapy in this status. Statins are common lipid-lowering drugs, which were wildly used in the clinic. But in recent years, it is reported that the sort of drug might lead to many adverse events.^[1]^ Acupoint catgut embedding, a novel therapy developed from acupuncture, stimulates acupoints by implanting protein line into the subcutaneous tissue to achieve therapeutic effects.^[12,13]^ It has been verified to be beneficial in individuals with Hyperlipidemia and obesity. Multiple studies have observed the amelioration of blood lipid and weight after the administration of acupoint catgut embedding.^[14,15]^ Even more fascinating, acupoint catgut embedding is only treated once every 2 weeks even more, which makes it more acceptable and attractive. But there is still no sufficient and strong testimony to testify it. Therefore, we design a meta-analysis to find out whether acupoint catgut embedding is definitely feasible in hyperlipidemia with obesity and provide reliable evidence for clinical application.

## 2 Methods and analysis

### 2.1 Registration

The protocol for this systematic review was registered on INPLASY and is available in full on the inplasy.com (https://inplasy.com/inplasy-2020-4-0036/). The registration number is INPLASY202040036 and the DOI number is 10.37766/INPLASY2020.4.0036. Additionally, this protocol report is structured according to the Preferred Reporting Items for Systematic Reviews and Meta-Analyses Protocols (PRISMA-P) statement guidelines.

### 2.2 Inclusion criteria of study selection

#### 2.2.1 Type of studies

The type of study is limited to randomized controlled trials (RCTs) carried out in humans. If the experiment shows that the phrase is random and the blind method is not limited, it will be taken into account as a random study. These following literary types are not taken into account: controlled (non-randomized) clinical trials, observational study, case reports, animal mechanism studies, self-controlled, random crossover studies.

#### 2.2.2 Types of participants

Participants who were both defined as hyperlipidemia and obesity are eligible to be included. The exclusion criteria are as follows: individuals who had accepted weight loss or Lipid-lowering therapy within 12 weeks before screening; women who were lactating or of childbearing potential.

#### 2.2.3 Types of interventions

The intervention group will use acupoint catgut embedding therapy, while the control group adopts a placebo, or no treatment, drugs (modern medicine or traditional Chinese medicine(TCM)), diet and exercise therapy, TCM therapies, other active treatments.

### 2.3 Outcomes

The primary outcomes are the lipid-lowing and wight-lowing effect (include reduction of serum TC, TG, LDL-C, HDL-C, as well as BMI, WB, WC from baseline to the end of studies). The second outcome is the number of adverse events during this period.

### 2.4 Study search

We will collect clinical studies by searching the following database: PubMed, Embase, Web of Science, CENTRAL, and 4 Chinese databases (involve CNKI, Wanfang, VIP, CBM) with a language restriction of English and Chinese until January 28, 2020. Together, to find additional potential studies, grey articles will be reviewed and reference lists of articles retrieved and related journals will be searched manually. The search terms included Hyperlipidemias, Hyperlipemia, Hyperlipemias, Hyperlipidemia, Lipidemia, Lipidemias, Lipemia, Lipemias, Dyslipidemias, Dyslipidemia, Dyslipoproteinemias, Dyslipoproteinemia, as well as obesity, Acupoint catgut embedding, catgut implantation, catgut embedding. The specific search strategy performed in Pubmed is presented in Table 1.

**Table 1:**
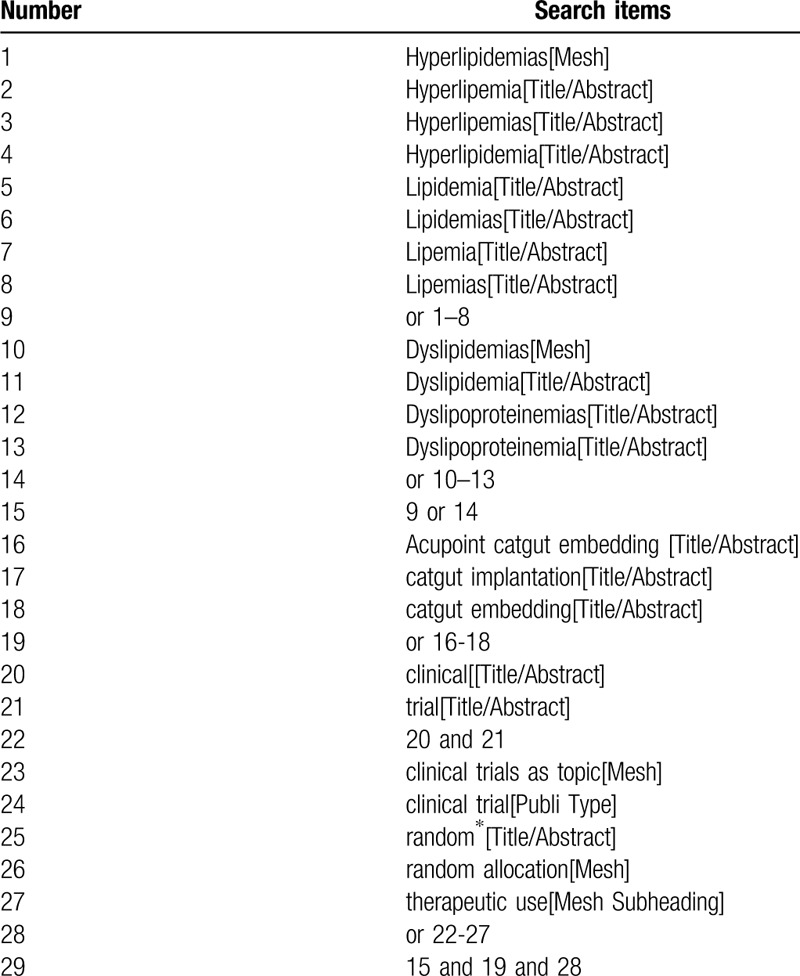
Search strategy in PubMed.

### 2.5 Study selection and analysis

#### 2.5.1 Study selection

**Figure 1:**
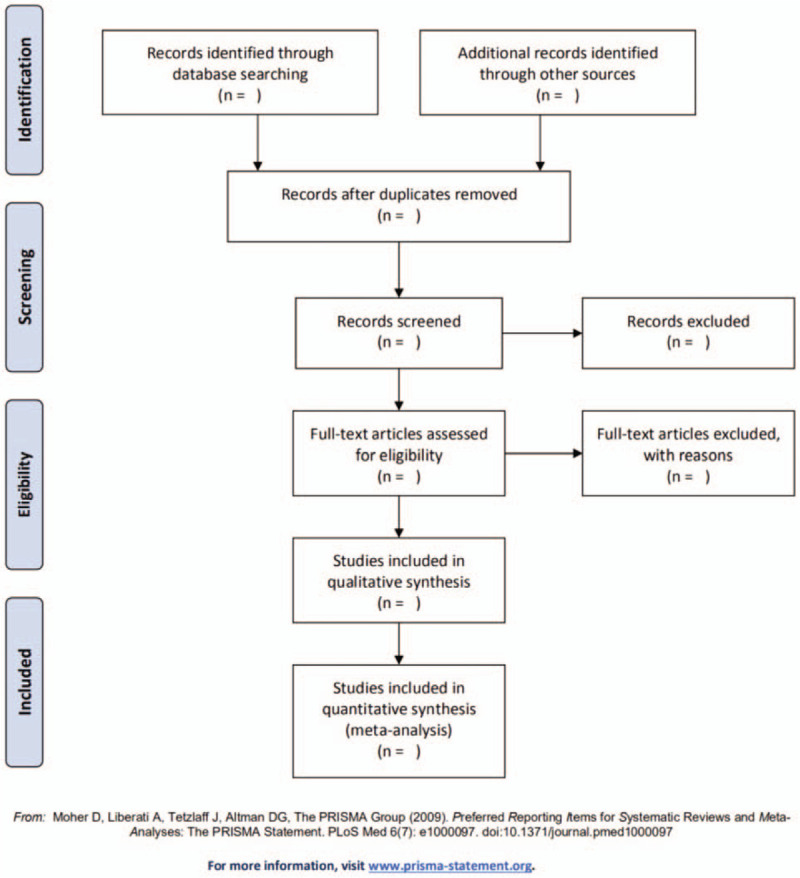
The PRISMA flow chart.

Document and date management will be done using Endnote X8. We will import all obtained studies into the software and perform the initial screen for eliminating inappropriate articles by inspecting all titles and abstracts; then, ultimate eligible articles can be selected after further reading the full-text of remainders. The number of excluded articles and reasons for exclusion will be recorded explicitly. The selection process will present in the PRISMA flow diagram (Fig. 1). All screening and selection process will be conducted separately with 2 investigators. If there is an argument, they will reach an agreement through discussion.

#### 2.5.2 Date extraction

Two independent investigators will carry out date extraction, and any conflicts in this process will be addressed by discussing with other reviewers. Dates extracted from each trial involve publication information, baseline characteristics (such as age, sex, race, disease history, etc.), sample size, intervention measure of trial and control group, status and duration of disease, outcomes (include lipide levels (LDL-c, HDL-c, TC, TG) and weight indexes (BMI, WC, WB, F%)), etc. Of course, we will also contact the original author if there is missing or insufficient information in the eligible article.

#### 2.5.3 Quality assessment

The Cochrane collaborations tool for accessing the risk of bias installed in RevMan V.5.3 will be used to investigate the quality of all selected trials. This accessing tool contains seven bias items, and each item is divided into 3 risk levels according to criteria. With this tool, 2 reviewers will access the risk level of each item in turn and independently judge the risk of bias of each trial. Then, the overall quality of the included studies will be presented at a glace through an automatically generated risk of bias graph or summary. Again, all disagreements will be settled with a discussion.

#### 2.5.4 Measures of treatment effect

We choose the RevMan V.5.3 to perform the statistical analysis of data. By convention, for continuous variables, we adopt the mean difference (MD) with 95% CIs to calculate the effect size. In addition, related risks (RRs) with 95% CIs will be applicable when it comes to dichotomous data.

#### 2.5.5 Assessment of heterogeneity

The heterogeneity can be implied through inspecting the forest plots and calculating *I*^2^ statistics. If *P* > .05 or *I*^2^ < 50%, the studies are homogeneous, and the fixed-effect model can be selected. Otherwise, it is interpreted as significant heterogeneity, and we will choose the random-effect model to execute date analysis. Furthermore, if significant heterogeneity is found in the meta-analysis, sensitivity analysis or subgroup analysis will be implemented as remedy to explore the sources of heterogeneity.

#### 2.5.6 Subgroup analysis

We will establish subgroup according to the different comparisons, or other factors that may affect outcomes, such as age, interval and duration of treatment, diagnostic criteria, quality of studies, etc.

#### 2.5.7 Sensitivity analysis

Furthermore, sensitivity analysis will be performed to evaluate the quality and stability of meta-analysis results. One analysis solution is to incorporate with random-effect model. On the other hand, we can exclude each included study 1 by 1 and re-analysis these dates to pinpoint the trial that induced distinction and finally eliminate it from eligible studies.

#### 2.5.8 Assessment of publication bias

A funnel plot generated in RevMan is taken to investigate potential publication bias, and the search may exist publication bias if the funnel plot is asymmetric.

### 2.6 Ethics and dissemination

The systematic review and meta-analysis do not require to pass the ethics approval. Because we include published articles rather than directly adopt interventions in participants. Ultimately, we will publish the results at a peer-reviewed journal follow as the review is completed.

## 3 Discussion

Hyperlipidemia, as one of the pathological bases of atherosclerosis, significantly increases the risk of cardiovascular and cerebrovascular diseases, stroke, as well as acute pancreatitis, which has represented a serious health hazard to the public. Hyperlipidemia with obesity is prevalent in the clinic, and obesity will further intensify adverse cardiovascular outcomes in patients with hyperlipidemia. Acupoint catgut embedding is an effective treatment for hyperlipidemia with obesity. However, systematic statistical evidence is still lacking. Thus, we intend to conduct a meta-analysis to investigate the therapeutic effect of acupoint catgut embedding in hyperlipidemia with obesity and provide more options for its clinical treatment.

Limited by languages, we just search articles written with English or Chinese, which may shrink the searching size. The interventions in control groups are various, which means significant heterogeneity during meta-analysis may be inevitable. To deal with this issue, the establishment of subgroups will hinge on different comparisons in the control group as well as other influencing factors.

## Author contributions

**Conceptualization:** Peipei Hong, Yang Gao, Qiu Chen.

**Data curation:** Qiuyue Wang, Xianliang Qiu.

**Investigation:** Qiuyue Wang, Xianliang Qiu.

**Methodology:** Peipei Hong, Yang Gao.

**Software:** Peipei Hong.

**Supervision:** Qiu Chen.

**Writing – original draft:** Peipei Hong, Yang Gao.

**Writing – review:** Qiu Chen.
